# Social networks and eating disorders during the Covid-19 pandemic

**DOI:** 10.1515/med-2021-0291

**Published:** 2021-08-24

**Authors:** Concetta M. Vaccaro, Giulia Guarino, Dario Conte, Emanuela Ferrara, Laura Dalla Ragione, Renata Bracale

**Affiliations:** Fondazione Censis, Health and Welfare Department, Piazza di Novella, 2, Rome, Italy; Department of Medicine and Sciences for Health, Molise University, Campobasso, Italy; Psychiatry and Rehabilitation Eating Disorders Department, USL1 Umbria, Umbria, Italy; Campus Biomedico University of Rome, Rome, Italy

**Keywords:** social network, ED, Covid-19, body shaming

## Abstract

**Objective:**

The purpose of this study is to assess the increase both in the use of the Internet and social media and in Google searches regarding eating disorders (ED) in Italy during the Covid-19 pandemic. Our aim is also to verify the possible impact of such an increase on ED, since patients treated for ED by the National Health Service (NHS) have increased in the first 6 months of 2020 as well.

**Method:**

We used data from Wearesocial surveys on Internet users in the first 6 months of 2020 and the Google searches related to the query of “food disorders” and “body shaming.” The first results of a project of the Italian Ministry of Health on ED have been considered too.

**Results:**

The social media users in July 2020 increased to 60% of the Italian population; a tendential increase in Google searches on these issues has emerged. Finally, new patients of NHS with ED showed a high increase in the first 6 months of 2020 (+40.9%).

**Conclusion:**

Considering the contents diffused on the Internet, it is fundamental to watch over net usage in the adolescent population and those with ED, because massive access to social media can be considered almost as a risk factor.

## 1 Introduction

Coronavirus 2 (SARS-COV-2) is responsible for acute respiratory syndrome; in March 2020, the World Health Organization (WHO) declared it a pandemic [1]. To avoid the diffusion of the virus and an increase in cases in Italy, the Government has approved the Decree-Law “I stay at home” [2]; it declared every type of public gathering is forbidden and thus closed many public and workplaces. Going out is allowed only for reasons of necessity. Consequently, digital platforms have offered a strategic opportunity for communicating with the outside world. Even before the lockdown, social media had been fundamental in our everyday lives (for example, Facebook, Instagram, Twitter, and YouTube) [3,4]. They are sources of information but also sharing tools [5] that have grown in importance due to the rarefaction of social interactions following the emergency. This is true also for the people affected by eating disorders (ED) who could have experienced a worsening of symptoms related to living alone. On the contrary, they could become very close to their families or, again, develop a change in their food habits [6,7].

Anorexia, bulimia, and binge eating are the three principal disorders, all connected to each other; the subject can migrate from one disorder to another [8]. The web can be also used as a tool for sharing different experiences in regards to different types of ED. Social networks can, in fact, amplify Body shaming [9]. More generally, during the lockdown, social networks have represented a tool through which people could shield themselves from a weight gain induced by their excessive sedentary lifestyle [6]. The aim of this study is that of evidencing the increase, during the lockdown, in the use of the Internet, in the time spent on social networks, and in the Google searches regarding ED in Italy during the Covid-19 pandemic. Our aim is also to verify its possible impact on these disorders, using as an indicator the increase of new patients treated by Public services for ED. In addition to the increased sedentary lifestyle, the contents of much of the online communication may have had a negative impact on the people who are most fragile and sensitive to this issue.

Together with the general data on Internet users, the data on Google searches related to food disorders in the same period have served to understand the trend of online searches on such issues. Finally, the data relating to patients with ED analyzed in a study of the National Center for Disease Prevention and Control (CCM) of the Italian Ministry of Health, concerning new patients treated by Public services for ED, confirm a relevant increase of them during the first months of lockdown [10].

## 2 Methods

This research used data from Wearesocial (results of GlobalWebIndex international surveys in different years among 1,010 Italian Internet users aged 16–64) on the increase in the number of Internets and social media users over the last years and particularly in the first 6 months of 2020 [5].

It added other data from a survey from the same source (GlobalWebIndex survey among samples of about 1,000 responses per country of Internet users aged 16–64 years), concerning the time spent on social networks by the Internet users of the principal European countries in the first months of 2020 due to the Covid-19 emergency. Moreover, it considered the frequency of some keywords related to food disorders in the Google searches in Italy between 4 August, 2019, and 26 July, 2020. Finally, we have analyzed the data of the CCM national study, by the Italian Ministry of Health, “Platform for fighting malnutrition in all its forms (triple burden: malnutrition by defect, by excess and by micro-nutrients).” The first part of the project concerning patients of public services for ED was coordinated by the Umbria region (scientific coordinator of the project Dr. Dalla Ragione) [10]. The observational study concerning patients obtained the approval of the ethics committee of Umbria Region (protocol CER (Comitato Etico Regionale) Umbria N.20032/20/ON of 19/11/2020). More specifically, among the objectives of the project, there was the realization of a national “survey” which collected activity data of all the services/districts/SIAN (food and nutrition hygiene services)/hospitals of the national territory dealing with malnutrition, as well as SDO (hospital discharge form) data relating to hospital admissions of such patients. The data were collected in an *ad hoc* platform and those reported are some first results, related to the first 6 months of 2020.

## 3 Results

All the analyses on web usage trends in Italy, in the last years, agree in highlighting its increase. Wearesocial data underline a 19% increase in net users between 16 and 64 years old among the total population from 01 January, 2016, to 01 January, 2020. In absolute value, we have switched from 37.63 million to 43.48 million people (82% of the total). Social media users have greatly increased from 47 to 58% (35 million). The average time of net usage per day has increased from a little more than 4 h to the current 6 h (Table 1).

**Table 1:** Internet and social media users in Italy (16–64 age) (absolute values and %)

	Internet users		Social media users		Average Internet usage time per day
	Absolute values in millions	(%)	Absolute values in millions	(%)	
1/1/2016	37.63	63	28.00	47	4H01M^†^
1/1/2017	39.21	66	31.00	52	N.A.
1/1/2018	43.21	73	34.00	57	6H08M
1/1/2019	54.80	92	35.00	59	6H04M
1/1/2020	43.48	82	35.00	58	6H00M

^†^Data referred to computer access (2H02M on mobile).

Source: Wearesocial data processing (GlobalWebIndex international surveys in different years among 1.010 Italian Internet users aged 16–64 years).

Furthermore, there has been an increase in Internet usage during the Covid-19 emergency [5]. The data on social network users of July 2020 have raised to 60% of the total population considered. A survey on Internet users in the first months of 2020 has shown that Italian Internet users who have affirmed to spend more time on social media due to the emergency are 43%, similar to Spain, compared to 31% in France and the UK and 19% in Greece (GlobalWebIndex survey among samples of about 1,000 responses per country of Internet users aged 16–64 years) [5].

**Figure 1:**
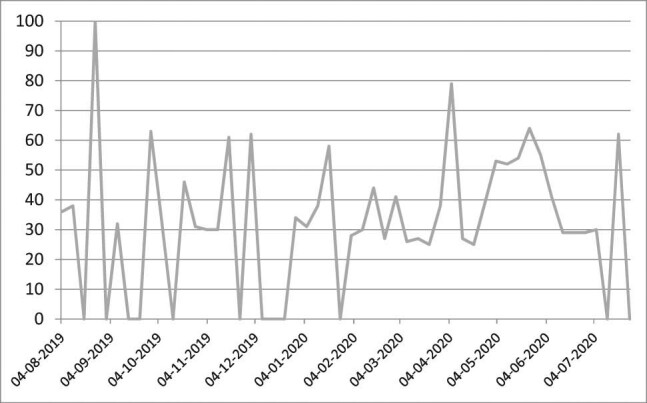
Frequency of Google searches of the query “ED” by Italian users (4/08/19–26/07/20) (*index numbers*). Source: Google trends.

The frequency of Google search queries on “ED” between 4 August, 2019, and 26 July, 2020, was hence assessed to verify the quantitative dimension and the variations in the interest of web users on the topic in the first months of 2020 (Figure 1).

**Figure 2:**
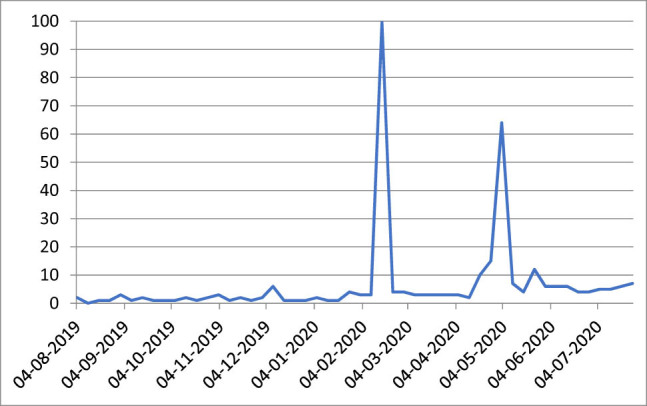
Frequency of Google searches of the query “body shaming” by Italian users (4/08/19–26/07/20) (*index numbers*). Source: Google trends.

Figure 1 highlights a quite fluctuating frequency that tends to stabilize, starting from February 2020, with peaks during the first months of 2020 until the end of July. The analysis of the search frequency of the query “body shaming” on Google has a much more clear-cut trend. Starting right from February, there are strong peaks in its searches on the net. Some increase in frequency is also present in the most recent of weeks, and the data are generally slightly on the rise (Figure 2).

Moreover, a general impact of the first lockdown period can be reflected in the 40.9% increase of new patients treated by public services for ED in the first 6 months of 2020 compared to the same period of 2019 (from 163.547 new patients in the first semester of 2019 to 230.458 in the first semester of 2020).

## 4 Discussion

This historical time is pervaded with anxiety, fear, and loss of control. The fear of contracting the illness can be associated with a loss of control, which people suffering from anorexia or bulimia try to compensate for by further increasing food restrictions. On the contrary, the same loss of control could trigger more frequent episodes of binge eating for those who suffer from such a disorder [6]. During the lockdown period, people being treated for ED, who may have taken months to find the courage to ask for help and embark on a path or may have waited months for hospitalization, have found themselves having to stop treatment or not being able to start it, increasing the risk of aggravation, chronicity, or relapse of the disorder [11].

Nevertheless, a significant increase in new patients has occurred, and this may be linked to the worsening of problems and/or emergencies.

The tedious household routine forces adolescents to spend the great majority of their time closed in their rooms while surfing the Internet. Wearesocial data from July 2020 underline that social media users have reached 60% of the total population. Furthermore, the Italian net users declared that they keep spending more and more time on social media during this period due to the emergency (43%). Our results have also shown that the Google search frequency of the query “ED” by Italian users is increasing despite fluctuations. Moreover, the search frequency of the query “body shaming” has shown an increase starting right from February with a first peak in the same month (100 in the chart of Google trends) and the second one in May. Some increase in frequency is also present in the last weeks, and data are generally slightly on the rise. While in the first case “ED,” there is a great number of searches directed to the gathering of information on this phenomenon (with some references also to local news), in the second, the search for information and the increase in communication are referred essentially to celebrity VIPs and their latest news. Adolescents have shown the tendency to use the Web not only as a leisure platform but also, more and more frequently, as an information tool [12]. Digital platforms are the fertile ground upon which to cultivate the myth of a slender and ideally perfect physique through the sharing of images, photos, and videos. This has led, directly or indirectly, to the spread of common stereotypes according to which overweight people are deemed lazy and lack self-control. At the same time, they help promote unrealistic physical appearances and extreme methods of weight control typical of nervous anorexia [13]. There are many examples of this tendency: Instagram and Facebook profiles with pictures of skinny bodies and phrases that praise resisting hunger. In this virtual reality where “thin is beautiful,” younger people who do not mirror this perspective are bound to be isolated from the masses and often bullied. There is a high risk of losing self-confidence and of interiorizing the idea that it is necessary to appear always as one’s perfect version. Adding up to this, virtual reality grows in importance over real life. As a consequence, physical appearance becomes the first thing to consider when relating with others [14]. In the most vulnerable subjects, adolescents, it can lead to the onset of ED.

Food becomes a means for handling emotions, especially during a global pandemic, when anxiety, stress, sadness, and loneliness have become constants in their everyday life [7]. On one hand, this means that social networks can serve as a tool to avoid complete isolation; on the other hand, if not utilized correctly, they can work as risk factors in the development of dysfunctional eating habits or pathologies with dire consequences on health.

It has never been as important as it is today, now that the Web has grown to be the principal instrument to feel close, despite being far, to watch over those who make the greatest use of these tools, the adolescents.

In fact, the new spread habits regarding web accessibility represent one of the key aspects relating to lifestyles that can impact health conditions, up to the point of becoming risk factors.

Families and schools must promote healthy and conscious use of the Internet, especially when dealing with the crucial theme of one’s physical perception.
